# Supplementary material for: Peaceful dying among Canada’s elderly: An analysis of the Canadian Longitudinal Study on Aging
Source: PLoS One. 2025 Jan 24;20(1):e0317014. doi: 10.1371/journal.pone.0317014 (PMC11760003; doi:10.1371/journal.pone.0317014)

Figure S1: Histogram of Time to Interview After Participant Death, Canadian Longitudinal Study on Aging, 2012-2022

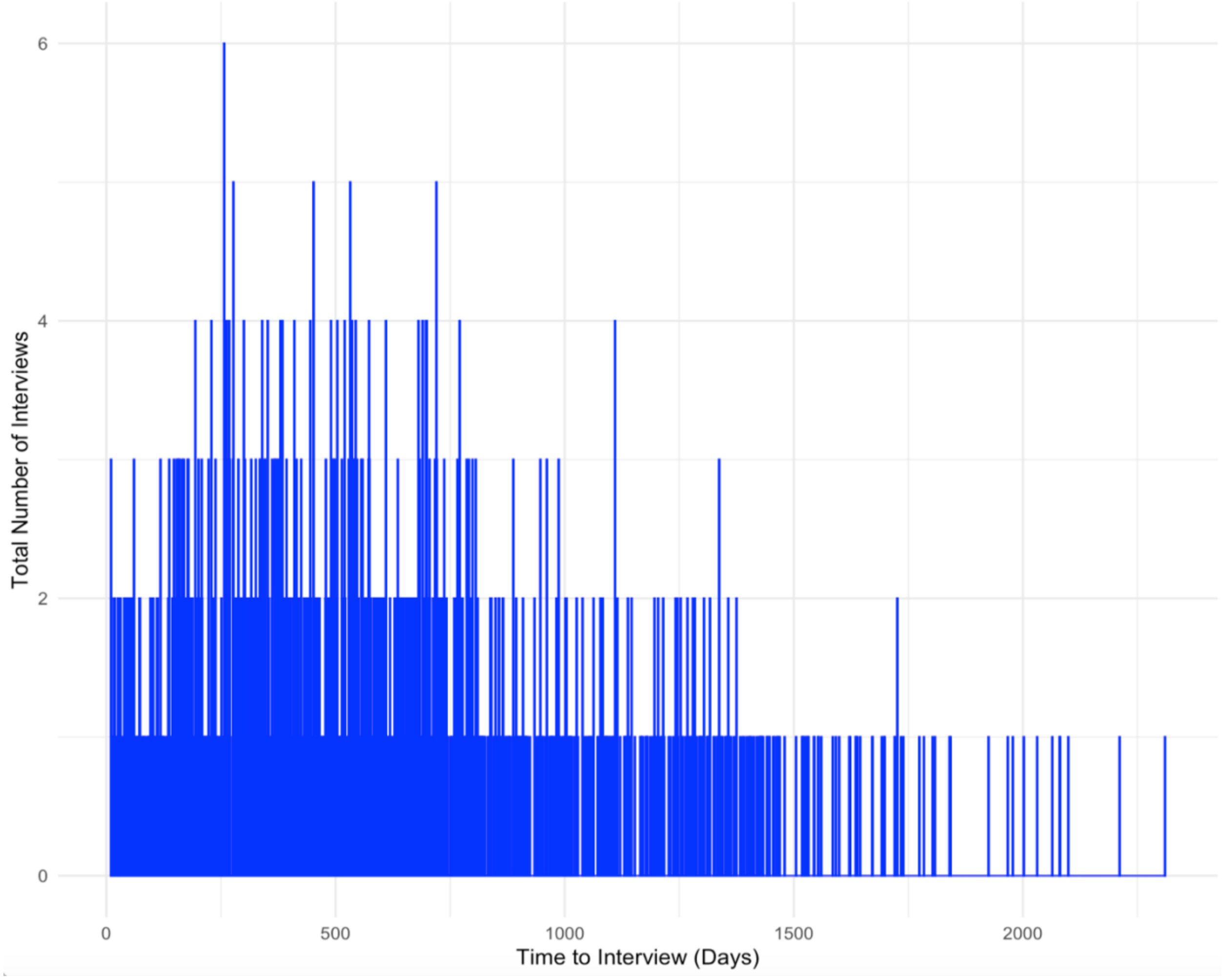

Supplement: S1 Fig — (PDF) [file pone.0317014.s001.pdf]
