# Supplementary material for: Peaceful dying among Canada’s elderly: An analysis of the Canadian Longitudinal Study on Aging
Source: PLoS One. 2025 Jan 24;20(1):e0317014. doi: 10.1371/journal.pone.0317014 (PMC11760003; doi:10.1371/journal.pone.0317014)
Supplement: S1 Table — (PDF) [file pone.0317014.s002.pdf]

Table S1: Characteristics of Deceased Canadian Longitudinal Study on Aging Participants with a Completed Decedent Interview (2012-2022) compared to Canadian Decedent Population (2012-2022)

**Participant Characteristics**	**Variable**	**CLSA [n(%)]**	**Canada [n(%)]**
**Total Deceased**		**1,287**	**2,936,807**
Sex	Male	798 (62.0)	1,481,701 (53.4)
	Female	489 (38.0)	1,291,489 (46.6)
Age at Death	45-64	116 (9.0)	470,365 (13.9)
	65-74	313 (24.3)	549,787 (18.7)
	75+	858 (66.7)	1,916,655 (67.4)
Marital Status	Single, never married or never lived with a partner	82 (6.4)	341,954 (12.1)
	Married/Living with a partner in a common-law relationship	807 (62.7)	1,150,610 (40.7)
	Widowed	261 (20.3)	1,045,606 (37.0)
	Divorced	115 (8.9)	135,015 (4.8)
	Separated	22 (1.7)	26,938 (1.0)
	Unknown	0 (0.0)	126,172 (4.4)
Location of death*	Hospital	631 (49.0)	1,831,105 (59.2)
	Non-Hospital	629 (48.9)	1,253,857 (40.5)
	Unknown	27 (2.1)	7,825 (0.3)
Cause of Death	Cancer	511 (39.7)	1,235,307 (42.1)
	Heart disease	33 (2.56)	644,241 (21.9)
	Respiratory disease**	51 (4.2)	110,572 (3.8)
	Dementia (e.g. Alzheimer's)	30 (2.5)	30,529 (1.0)
	Accident	32 (2.6)	103,978 (3.5)
	Influenza or pneumonia	43 (3.5)	60,762 (2.1)
	Suicide	8 (0.7)	21,292 (0.7)
	Kidney disease***	25 (2.1)	46,660 (1.6)
	Other	554 (43.0)	684,276 (23.3)

*Values reported for entire Canadian Population

**Respiratory diseases including emphysema, obstructive lung disease, asthma, chronic obstructive pulmonary disease

***Kidney Diseases such as nephritis, nephrotic syndrome, or nephrosis
