# Supplementary material for: Peaceful dying among Canada’s elderly: An analysis of the Canadian Longitudinal Study on Aging
Source: PLoS One. 2025 Jan 24;20(1):e0317014. doi: 10.1371/journal.pone.0317014 (PMC11760003; doi:10.1371/journal.pone.0317014)
Supplement: S2 Table — (PDF) [file pone.0317014.s003.pdf]

Table S2: Correlation Matrix for all Participant Characteristics and End-of-Life Characteristics, Canadian Longitudinal Study on Aging, 2012-2022

Participant Characteristics	Sex	Age	Ethnicity	Religion	Education	Marital Status	ADL/ IADL	Final Caregiver	Health POA***	EoL POA***	Closeness	Last Doctor Visit	Cause of Death	Location of Death
Sex	1.00	-0.045	0.013	0.068	-0.010	0.121	0.021	0.089	0.076	0.054	0.002	-0.003	-0.040	0.028
Age	-0.035	1.000	0.018	0.070	-0.083	0.285	0.072	0.228	0.078	0.075	-0.024	0.033	0.073	0.054
Ethnicity	0.013	0.018	1.000	0.012	-0.062	0.017	-0.014	0.019	0.023	-0.013	-0.021	0.030	-0.062	-0.013
Religion	0.068	0.070	0.012	1.000	-0.175	0.055	0.068	0.013	0.026	0.022	-0.009	0.007	-0.012	-0.001
Education	-0.010	-0.083	-0.062	-0.175	1.000	-0.077	0.022	-0.056	0.027	0.023	-0.016	-0.038	-0.021	-0.014
Marital Status	0.121	0.285	0.017	0.055	-0.077	1.000	0.093	0.239	0.124	0.084	0.108	0.000	0.027	-0.025
ADL/IADL*	0.021	0.072	-0.014	0.068	0.022	0.093	1.000	0.104	0.192	0.127	0.048	-0.131	-0.027	0.230
Final Caregiver	0.089	0.228	0.019	0.013	-0.056	0.239	0.104	1.000	0.171	0.140	0.061	-0.033	0.035	0.038
Health SDM**	0.076	0.078	0.023	0.026	0.027	0.124	0.192	0.171	1.000	0.527	0.128	-0.095	-0.010	0.055
EoL SDM***	0.054	0.075	-0.013	0.022	0.023	0.084	0.127	0.140	0.527	1.000	0.096	-0.114	0.010	0.020
Close to Deceased	0.002	-0.024	-0.021	-0.009	-0.016	0.108	0.048	0.061	0.128	0.096	1.000	-0.038	0.032	-0.068
Last Doctor Visit	-0.003	0.033	0.030	0.007	-0.038	0.000	-0.131	-0.033	-0.095	-0.114	-0.038	1.000	-0.010	-0.085
Cause of Death	-0.040	0.073	-0.062	-0.012	-0.021	0.027	-0.027	0.035	-0.010	0.010	0.032	-0.010	1.000	0.022
Location of Death	0.028	0.054	-0.013	-0.001	-0.014	-0.025	0.230	0.038	0.055	0.020	-0.068	-0.085	0.022	1.000

*ADL/IADL=Activities of Daily Living/ Instrumental Activities of Daily Living

**Health SDM=End-of-life Substitute Decision Maker

***EoL SDM=End-of-life Substitute Decision Maker
