# Supplementary material for: Peaceful dying among Canada’s elderly: An analysis of the Canadian Longitudinal Study on Aging
Source: PLoS One. 2025 Jan 24;20(1):e0317014. doi: 10.1371/journal.pone.0317014 (PMC11760003; doi:10.1371/journal.pone.0317014)
Supplement: S3 Table — (PDF) [file pone.0317014.s004.pdf]

Table S3: Variance Inflation Factor for all Participant Characteristics and End-of-Life Characteristics, Canadian Longitudinal Study on Aging, 2012-2022

**Participant Characteristics**	**VIF**	**Degrees of Freedom**
Sex	1.24	1
Age	1.28	2
Ethnicity	1.03	1
Religion	1.06	1
Education	1.13	3
Marital Status	2.36	2
ADL/IADL*	1.31	3
Final Caregiver	2.33	2
Healthcare Arrangements	1.49	1
End-of-Life Arrangements	1.42	1
Closeness	1.07	1
Last Doctor Visit	1.35	4
Cause of Death	1.41	3
Location of Death	1.59	3

*ADL/IADL=Activities of Daily Living/ Instrumental Activities of Daily Living
