# Supplementary material for: Peaceful dying among Canada’s elderly: An analysis of the Canadian Longitudinal Study on Aging
Source: PLoS One. 2025 Jan 24;20(1):e0317014. doi: 10.1371/journal.pone.0317014 (PMC11760003; doi:10.1371/journal.pone.0317014)
Supplement: S4 Table — (PDF) [file pone.0317014.s005.pdf]

Table S4: End-of-Life Characteristics for Males and Females With Completed Decedent Interviews, Canadian Longitudinal Study on Aging, 2012-2022

Variable Description	Did Not Experience Peace with Dying	Did Not Experience Peace with Dying
**Primary cause of death**	**Females n(%)**	**Males n(%)**
Cancer	154 (46.7)	220 (41.9)
Heart disease	70 (21.2)	128 (38.8)
RIDK*	25 (7.5)	60 (11.4)
Other	81 (24.5)	117 (22.3)
**Location of Death**		
Their own home	68 (20.6)	119 (22.7)
Other private home	7 (2.1)	1 (0.2)
Residence for seniors	9 (2.7)	3 (0.6)
Hospital	147 (44.5)	253 (48.2)
Hospice	25 (7.6)	35 (6.7)
Palliative care unit	41 (12.4)	64 (12.2)
Senior Home or LTC Facility**	28 (8.5)	40 (7.6)
Other (please specify)	5 (1.5)	10 (1.9)
**Alternative Health Care Decision Maker**		
Yes	288 (87.3)	431 (82.1)
No	42 (12.7)	94 (17.9)
**Alternative End-of-Life Care Decision Maker**		
Yes	272 (82.4)	403 (76.8)
No	58 (17.6)	122 (23.2)

*RIDK=R=Respiratory diseases including emphysema, obstructive lung disease, asthma, chronic obstructive pulmonary disease; I=Influenza or pneumonia; D=Dementia; K=Kidney Diseases such as nephritis, nephrotic syndrome, or nephrosis

**LTC=Long-term Care
