# Supplementary material for: Peaceful dying among Canada’s elderly: An analysis of the Canadian Longitudinal Study on Aging
Source: PLoS One. 2025 Jan 24;20(1):e0317014. doi: 10.1371/journal.pone.0317014 (PMC11760003; doi:10.1371/journal.pone.0317014)
Supplement: S5 Table — (PDF) [file pone.0317014.s006.pdf]

Table S5: Subgroup Analysis of Canadian Longitudinal Study on Aging Deceased Participants with a Completed Decedent Interview Based on Cause of Death, 2012-2022

**Variable Category**	**Variable Characteristic**	**Cancer**	**Non-Caner**
		**Unadjusted OR(95%CI)**	**Unadjusted OR(95%CI)**
**Sex**	Female	Reference	Reference
	Male	0.76 (0.49-1.12)	1.34 (0.94-1.9)
**Age**	45-64	Reference	Reference
	65-74	1.32 (0.77-2.24)	1.01 (0.61-1.66)
	75+	1.53 (0.91-2.53)	1.21 (0.75-1.94)
**Ethnicity**	Non-White	Reference	Reference
	White	2.77 (0.51-15.12)	1.17 (0.41-3.18)
**Religion**	No Religious Beliefs	Reference	Reference
	Holds Religious Beliefs	1.24 (0.76-1.99)	1.35 (0.9-2.02)
**Education**	Less than High School	Reference	Reference
	High School	1.60 (0.67-3.92)	0.8 (0.45-1.44)
	Other post-secondary education	1.00 (0.51-1.90)	0.81 (0.5-1.3)
	University degree or above	1.04 (0.53-1.99)	0.72 (0.44-1.17)
**Marital**	Married	Reference	Reference
	Single/Divorced	0.54 (0.33-0.89)	1.04 (0.62-1.75)
	Widowed	1.77 (0.98-3.42)	1.58 (0.96-2.61)
**ADL & IADL***	No/Mild Impairment	Reference	Reference
	Moderate impairment	0.80 (0.43-1.51)	2.00 (1.16-3.55)
	Severe/Total Impairment	0.65 (0.40-1.06)	1.45 (1.00-2.11)
**Caregiver**	Child	Reference	Reference
	Other	0.85 (0.48-1.49)	1.16 (0.76-1.8)
	Spouse	0.98 (0.59-1.59)	0.97 (0.6-1.55)
**Health Decision Making SDM****	Absent	Reference	Reference
	Present	**2.38 (1.47-3.82)**	0.72 (0.44-1.16)
**EoL Decision Making SDM****	Absent	Reference	Reference
	Present	**2.02 (1.30-3.12)**	1.77 (1.16-2.71)
**Closeness**	Not Close to Deceased	Reference	Reference
	Close to Deceased	1.06 (0.51-2.08)	1.58 (0.91-2.72)
**Last physician visit**	Did Not See Doctor Before Passing	Reference	Reference
	1-2 weeks	1.63 (0.86-3.33)	1.02 (0.64-1.65)

	3-6 Weeks	0.74 (0.36-1.50)	1.05 (0.63-1.77)
	7-51 Weeks	1.00 (0.48-2.24)	0.64 (0.39-1.05)
	52+ Weeks	0.77 (0.38-1.65)	0.97 (0.58-1.64)
**Location of Death**	Hospital	Reference	Reference
	Home	**1.19 (1.04-4.02)**	1.06 (0.72-1.58)
	Hospice or Palliative Care	1.42 (0.91-2.23)	1.33 (0.68-2.69)
	Senior/LTC[1]/Other	0.89 (0.38-2.17)	1.31 (0.81-2.14)

*ADL/IADL=Activities of Daily Living/ Instrumental Activities of Daily Living
** SDM=Substitute Decision Maker
***RIDK=R=Respiratory diseases including emphysema, obstructive lung disease, asthma, chronic obstructive pulmonary disease; I=Influenza or pneumonia; D=Dementia; K=Kidney Diseases such as nephritis, nephrotic syndrome, or nephrosis
[1]LTC=Long-term Care
