# Supplementary material for: Peaceful dying among Canada’s elderly: An analysis of the Canadian Longitudinal Study on Aging
Source: PLoS One. 2025 Jan 24;20(1):e0317014. doi: 10.1371/journal.pone.0317014 (PMC11760003; doi:10.1371/journal.pone.0317014)
Supplement: S6 Table — (PDF) [file pone.0317014.s007.pdf]

***Table S6*: Sensitivity Analysis of Peace with Dying for Participants with Completed Decedent Interview within One Year of Death Compared to More than One Year After Death, Canadian Longitudinal Study on Aging, 2012-2022

Variable Category	Variable Characteristic	Decedent Interview Completed within One Year of Death (n=395)	Decedent Interview Completed within More than One Year of Death (n=893)
		**Unadjusted OR(95%CI)**	**Unadjusted OR(95%CI)**
**Sex**	Female	Reference	Reference
	Male	1.63 (1.06-2.49)	0.72 (0.54-0.97)
**Age**	45-64	Reference	Reference
	65-74	1.21 (0.65-2.26)	1.13 (0.74-1.73)
	75+	1.49 (0.84-2.64)	1.31 (0.89-1.94)
**Ethnicity**	Non-White	Reference	Reference
	White	0.43 (0.05-3.91)	1.91 (0.77-4.74)
**Religion**	No Religious Beliefs	Reference	Reference
	Holds Religious Beliefs	1.92 (1.13-3.24)	1.07 (0.74-1.53)
**Education**	Less than High School	Reference	Reference
	High School	0.85 (0.36-2.00)	1.06 (0.60-1.86)
	Other post-secondary education	0.88 (0.44-1.75)	0.87 (0.56-1.37)
	University degree or above	1.00 (0.50-2.01)	0.75 (0.48-1.18)
**Marital**	Married	Reference	Reference
	Single/Divorced	0.62 (0.36-1.08)	0.78 (0.54-1.13)
	Widowed	1.12 (0.64-1.94)	1.73 (1.18-2.55)
**ADL & IADL***	No/Mild Impairment	Reference	Reference
	Moderate impairment	1.70 (0.88- 3.30)	1.72 (1.06-2.79)
	Severe/Total Impairment	1.42 (0.91-2.21)	1.33 (0.97-1.83)
**Caregiver**	Child	Reference	Reference
	Other	1.00 (0.57-1.77)	0.84 (0.57-1.23)
	Spouse	1.14 (0.69-1.88)	0.82 (0.58-1.15)
**Health Decision Making SDM****	Absent	Reference	Reference
	Present	1.69 (0.95-3.01)	1.65 (1.18-2.31)
	Absent	Reference	Reference

**EoL Decision Making SDM****	Present	1.62 (0.98- 2.67)	1.91 (1.41-2.60)
**Closeness**	Not Close to Deceased	Reference	Reference
	Close to Deceased	1.73 (0.84-3.53)	1.27 (0.78-2.07)
**Last physician visit**	Did Not See Doctor Before Passing	Reference	Reference
	1-2 weeks	1.13 (0.62-2.03)	1.06 (0.67-1.67)
	3-6 Weeks	0.85 (0.44-1.63)	0.87 (0.54-1.40)
	7-51 Weeks	0.82 (0.41-1.63)	0.56 (0.36- 0.89)
	52+ Weeks	0.42 (0.19-0.94)	0.90 (0.57-1.42)
**Cause of death**	Heart Disease	Reference	Reference
	Cancer	1.03 (0.58-1.81)	1.22 (0.82-1.82)
	Other	1.48 (0.87-2.51)	1.80 (1.26-2.59)
	RIDK***	0.88 (0.44-1.77)	0.81 (0.50-1.31)
**Location of Death**	Hospital	Reference	Reference
	Home	1.15 (0.68-1.93)	1.17 (0.82-1.66)
	Hospice/Palliative Care	1.88 (0.98-3.61)	1.57 (1.05-2.35)
	Senior/LTC[1]/Other	1.19 (0.61- 2.33)	1.16 (0.72-1.86)

*ADL/IADL=Activities of Daily Living/ Instrumental Activities of Daily Living
** SDM=Substitute Decision Maker
***RIDK=R=Respiratory diseases including emphysema, obstructive lung disease, asthma, chronic obstructive pulmonary disease; I=Influenza or pneumonia; D=Dementia; K=Kidney Diseases such as nephritis, nephrotic syndrome, or nephrosis
[1]LTC=Long-term Care
