# Supplementary material for: Peaceful dying among Canada’s elderly: An analysis of the Canadian Longitudinal Study on Aging
Source: PLoS One. 2025 Jan 24;20(1):e0317014. doi: 10.1371/journal.pone.0317014 (PMC11760003; doi:10.1371/journal.pone.0317014)
Supplement: S7 Table — (PDF) [file pone.0317014.s008.pdf]

Table S7: Sensitivity Analysis of Peace with Dying for Participants with Different Decedent Respondents, Canadian Longitudinal Study on Aging, 2012-2022

Variable Category	Variable Characteristic	Spouse (n=542)	Child (n=434)	Other (n=311)
		**Unadjusted OR(95%CI)**	**Unadjusted OR(95%CI)**	**Unadjusted OR(95%CI)**
**Sex**	Female	Reference	Reference	Reference
	Male	0.86 (0.52-1.41)	1.25 (0.87-1.81)	1.25 (0.71-2.24)
**Age**	45-64	Reference	Reference	Reference
	65-74	1.42 (0.85-2.37)	0.94 (0.52-1.7)	0.93 (0.43-1.96)
	75+	1.18 (0.71-1.95)	1.36 (0.76-2.44)	1.53 (0.73-3.19)
**Ethnicity**	Non-White	Reference	Reference	Reference
	White	1.01 (0.23-3.99)	1.07 (0.3-3.53)	3.12 (0.45-26.18)
**Religion**	No Religious Beliefs	Reference	Reference	Reference
	Holds Religious Beliefs	1.12 (0.7-1.78)	1.28 (0.78-2.08)	1.84 (0.84-3.99)
**Education**	Less than High School	Reference	Reference	Reference
	High School	0.81 (0.36-1.83)	1.22 (0.64-2.34)	1.12 (0.35-3.61)
	Other post-secondary education	0.56 (0.28-1.06)	1.14 (0.68-1.9)	0.81 (0.33-1.89)
	University degree or above	0.61 (0.31-1.16)	0.97 (0.56-1.65)	1.0 (0.39-2.44)
**Marital**	Married	Reference	Reference	Reference
	Single/Divorced	1.06 (0.22-5.87)	0.63 (0.39-1.04)	0.88 (0.4-1.88)
	Widowed	1.19 (0.2-9.7)	1.38 (0.86-2.22)	1.45 (0.61-3.43)
**ADL & IADL***	No/Mild Impairment	Reference	Reference	Reference
	Moderate impairment	1.32 (0.73-2.45)	1.25 (0.71-2.26)	0.57 (0.30-1.08)
	Severe/Total Impairment	1.46 (0.95-2.25)	0.79 (0.53-1.18)	0.91 (0.28-3.33)
**Caregiver**	Child	Reference	Reference	Reference
	Other	1.81 (0.33-9.0)	1.16 (0.77-1.76)	0.7 (0.25-1.79)
	Spouse	1.24 (0.2-6.99)	0.89 (0.5-1.59)	0.56 (0.14-2.07)
**Health Decision Making SDM****	Absent	Reference	Reference	Reference
	Present	1.25 (0.72-2.14)	0.97 (0.56-1.65)	1.08 (0.51-2.29)

**EoL Decision Making SDM****	Absent	Reference	Reference	Reference
	Present	1.35 (0.81-2.25)	2.03 (1.3-3.16)	1.51 (0.76-2.99)
**Closeness**	Not Close to Deceased	Reference	Reference	Reference
	Close to Deceased	2.35 (0.85-6.64)	1.05 (0.61-1.76)	0.75 (0.34-1.61)
**Last physician visit**	Did Not See Doctor Before Passing	Reference	Reference	Reference
	1-2 weeks	0.89 (0.5-1.64)	1.21 (0.71-2.1)	1.49 (0.62-3.93)
	3-6 Weeks	0.59 (0.31-1.12)	1.19 (0.65-2.24)	0.82 (0.33-2.14)
	7-51 Weeks	0.58 (0.31-1.09)	0.75 (0.41-1.38)	0.46 (0.18-1.15)
	52+ Weeks	0.81 (0.39-1.7)	0.99 (0.56-1.77)	0.35 (0.15-0.82)
**Cause of death**	Heart Disease	Reference	Reference	Reference
	Cancer	0.87 (0.50-1.50)	1.47 (0.91-2.4)	0.77 (0.35-1.63)
	Other	1.47 (0.86-2.49)	2.01 (1.26-3.24)	0.83 (0.32-2.16)
	RIDK***	0.58 (0.28-1.21)	0.81 (0.46-1.43)	1.02 (0.46-2.29)
**Location of Death**	Hospital	Reference	Reference	Reference
	Home	1.27 (0.78-2.09)	1.34 (0.83-2.18)	1.17 (0.56-2.51)
	Hospice/Pall	1.48 (0.84-2.67)	1.11 (0.65-1.9)	1.83 (0.81-4.29)
	Senior/LTC[1]/Other	1.18 (0.58-2.43)	1.56 (0.85-2.93)	1.74 (0.72-4.54)

*ADL/IADL=Activities of Daily Living/ Instrumental Activities of Daily Living
** SDM=Substitute Decision Maker
***RIDK=R=Respiratory diseases including emphysema, obstructive lung disease, asthma, chronic obstructive pulmonary disease; I=Influenza or pneumonia; D=Dementia; K=Kidney Diseases such as nephritis, nephrotic syndrome, or nephrosis
[1]LTC=Long-term Care
